# Supplementary material for: Knowledge, preventive behaviors and risk perception of the COVID-19 pandemic: A cross-sectional study in Turkish health care workers
Source: PLoS One. 2021 Apr 9;16(4):e0250017. doi: 10.1371/journal.pone.0250017 (PMC8034719; doi:10.1371/journal.pone.0250017)
Supplement: Attachment — Submitted filename: Reviewers comments.docx [file pone.0250017.s002.docx]

**Reviewers comments**

Thank you for allowing me to review this manuscript. This is an important study from Turkey Healthcare workers but the authors will need to reconsider their analysis and result presentation in a more robus scientific way. They need to do the extra work to turn the cursory descriptive analysis into a scientific analysis that keeps up against academic standards.

The problem is that the Authors purport to use statistical inference but their sample is totally biased, meaning that all inference is completely meaningless if not taking serious steps in controlling for bias, refining the population the Authors infer to and keeping interpretations within the realm of relationships between variables rather than characteristics of the population. More sophisticated statistical approaches and substantial analysis to control for biased data is needed

Instead of presenting coefficients for five variables (which is taking an inference to the population), the Authors should investigate what relationships hold when controlled for the other variables available. This can adjust for bias. Also, the Authors should reflect upon what relationships do make sense and interpret what they find regarding such reflections.

The Authors do not report which variables were included in the multiple regression in which they exclude insignificant variables. First, this approach can be criticised as significance hunting. Statistical significance is rather unimportant as it depends on sample size. Control for variables influencing participation in the survey and not predicting the outcome.

The outcome of knowledge, attitude and practice should be modelled separately to identify the associated factors stating the reasons behind the adjustments factors. Currently the use of preventive behaviour score is not inline with the study aims or title.

The study is not representative and front line workers as used in the paper is incorrect as this includes cleaners, security men in the hospital etc. Authors should stick to health care professionals. Surprisingly the high response rate of 98.3% is very doubtful going by the fact that the authors stated that survey was rolled out to as many people as possible’ (page 4). How did the authors keep track of the number of online surveys distributed (n=305) if they had used a link sent to people?

Discussion is a repeat of the introduction. However, it should summarise the study findings, compare those with current evidence and give reasons for where results differ. AUthors should seek for help in writing the discussion scientifically.

Besides controlling for variables measuring selection bias, interaction terms should be investigated. What happens if one crosses sex and region or education and region in predicting knowledge, attitude, and practice. Your models assume constant relationships across all variables. That is not necessarily the case and the value of the study cannot be to make statements about the population. But the value of the study is to explore potential relationships that are later to be verified by a random sample.

Calculate a multivariate model including *all theoretically meaningful* covariates that reflect selection bias and report odds ratios when controlling for the other variables. For example, education, sex, age etc. need to be included to calculate the odds ratios. This way, the sample’s bias for education, sex and age etc. will have less influence on the estimate of the outcomes as the estimates are reported when all other variables held constant

Describe the sample more clearly, survey distribution, how was it translated back to English, how did validation confir that meaning was not lost in translation?

Clear description and reflection on concepts used. Why were the variables included. Also, describe clearly what the variables „perception“ and „attitude“ mean. perception and attitude aren’t really mentioned n the text although the title reads so. The reader does not learn how they were measured. Attitude“ in itself does not mean anything. My attitude toward covid might be ignorant, nonchalant, fear-related, scientific, curiosity, whatever. It needs to be clearly named.

Present the results in more standard form, not variable by variable but present the whole model, including model statistics and N (item nonresponse is also an interesting point the Authors are tacit about).

Reflect more seriously about the bias of the sample: If you would use the same data to predict the knowledge in other regions in Turkey, how correct will you be? This gives a hint how correct you might be with the estimate.
