# Supplementary material for: Knowledge, preventive behaviors and risk perception of the COVID-19 pandemic: A cross-sectional study in Turkish health care workers
Source: PLoS One. 2021 Apr 9;16(4):e0250017. doi: 10.1371/journal.pone.0250017 (PMC8034719; doi:10.1371/journal.pone.0250017)
Supplement: Attachment — Submitted filename: Response.docx [file pone.0250017.s003.docx]

**24.10.2020**

**Dear Editor,**

We would like to thank you and the reviewers for their comments and recommendations. Based on these, we have revised our manuscript, and they have helped us improve its scientific quality. Unfortunately, due to majör revision, we rewrite the manuscript; therefore, we did not highlight the whole changes.

COMMENTS:
***Reviewer #1**

**Comment:** The title of the article is not grammatically correct. Overall, substantive editing will be needed to address grammar, spelling, and syntax. The methods are not clear and require substantial improvements, the tables and order of reported information in the results might support a better understanding of the results, and both the introduction and discussion are confusing to read; primarily because of superfluous information that in no way relate to the topic or findings of the study.

Introduction: The context and scope are not clearly defined in the manuscript. The first two paragraphs and the introduction in general do not align well with the scope of the paper.

Methods: The methodology is unclear, was it a snowball sampling approach? Is Google Form used in other studies? Please report and cite how effective Google Form is and what protections there are to avoid double counting and ensure that people who are not HCWs are taking the survey. In demographics, can you clarify "the current status"? For the COVID-19 related knowledge questions, were these yes/no options or true/false questions. This is later defined in the results but should be adequately described in the methods section. The Cronbach's alpha reported is quite low. This makes sense because you are measuring different domains. As you report the findings, it is unclear what these items actually measure. The composite score is not a single factor and therefore doesn't really make sense to assess this way. Werethere differences observed between domains?

Results: Table 1 should not include composite scores unless the instrument is better described in the methods. There is little information about what is included in the "knowledge score" or "preventative behaviors score". Given that the details of these composite items are not outlined is difficult for me to understand exactly what the outcome is measuring (as it is written in Table 1). Table 2 and 3 are more informative and define the items – this is great, but comes too late in the manuscript to help follow the first portion of the results section. However, given that this is the first mention of the items it is confusing to read in the order it is placed. Consider updating the methods to better provide information about the items included in the analysis.

Discussion: Line 218 to 227 don't have much value and contribute very little to this topic. There have been updated guidelines formask use, please update the citation and information. The section that discusses use of face coverings and other masks don't directly relate to the findings and seem out of place. Agreement to use one does not speak to efficacy and guidance of use. Paragraph between lines 270-279 are out of place and not relevant to the findings.

**Author response:** Regarding this comment, the methodology of the manuscript was evaluated, and the introduction, material methods, results were rewritten. Following that, we utilized an editing service to control and check the manuscript's typographical or grammatical errors. We also uploaded the editing service text check certificate as a separate file.

**Reviewer #2:**

**Comment:** The authors have tried to assess the knowledge and preventive behaviors of health care workers (HCWs) in Turkey. However, the work requires major corrections and I will suggest the work be given o professional English language editors.

My concerns:

The population of HCWs inTurkey is 1,016,401 (Anadolu Agency, 2020). Do you think 305 respondents is representative of this population?

Could you kindly educate me on how you calculated the statistical power for the validity of a survey instrument and got 51 responses?

Did you calculate the odds ratio in the multivariable logistic regression?

How did you grade the responses? Who (or which group) had poor, average or satisfactory knowledge and behavior among your respondents?

There was no mention of practices in result (So, I suggest you remove practices from the topic).

What is the minimum threshold for reliability of variable in a survey instrument (using Cronbach's alpha)?

**Author Response:** Under this comment, as mentioned above, the manuscript was rewritten and has been revised by a native English speaker from the editing service. Moreover, the study population was adjusted as requested. The sample size was calculated by power analysis using the G * Power 3.1.9.2 program based on gender groups in the publication. A professional statistician reevaluated all the statistical data and results.

**Reviewer #3:**

**Comment:** I have reviewed the manuscript titled "Knowledge, Attitudes and Practices of Turkish Healthcare WorkersAbout SAR2-CoV-19 During Pandemic" submitted to "PlosOne" for publication. In this paper, authors have conducted a survey study to investigate the level of COVID-19 related knowledge in health care workers in Turkey. This is a well conducted study and the manuscript is well presented and fits within the scope of the journal; it needs some major improvements; there are a few suggestions that authors may consider improving it further: The use of English language is reasonable, however, there are a number of punctuation and grammatical errors; that should be corrected and rephrased using academic English for a better flow of text for reader. Authors may consider proofreading this manuscript.

**Author response:** We would like to thank the reviewer for this specific comment; we rechecked and corrected the language errors with the proofreading.

**Reviewer # 4:**

**Comment:** The authors evaluated personality variables, specific concerns, proposeto self-isolate, and personal safety among healthcare workers practicing in Turkey during the current COVID-19 pandemic. The study is interesting and needs minor revision.

**Author response:** Thank you for this comment. In this manuscript, we aimed to demonstrate the Turkish Healthcare Workers, the frontline fighters in this pandemic. In our view, with this kind of manuscript, the healthcare workers would increase the perspective of this pandemic and also gain valuable experiences.

**Reviewer # 5:**

**Comment:** Study is flawed by the methodology, science behind the study, analysis and discussion was largely a repeat of the introduction. I have provided detailed guideline on what authors can do to improve the study if they wish to

**Author response:** Unnecessary information was excluded, and the introduction was rewritten. The methodology of the manuscript was corrected. In light of the results of the data, we rewrite the discussion part again.

**Special response:** As our reviewer comments on the manuscript were extensive, the manuscript has changed accordingly. Therefore, highlighting the changes in the manuscript was not possible.

On behalf of all of the authors, I would like to thank the reviewers for their valuable comments and recommendations.

Thank you for your attention.
