# Supplementary material for: Knowledge, preventive behaviors and risk perception of the COVID-19 pandemic: A cross-sectional study in Turkish health care workers
Source: PLoS One. 2021 Apr 9;16(4):e0250017. doi: 10.1371/journal.pone.0250017 (PMC8034719; doi:10.1371/journal.pone.0250017)
Supplement: Attachment — Submitted filename: Reviewers comments 2.docx [file pone.0250017.s004.docx]

**Reviewers comments**

I thank the authors for attempting to address the comments raised. However, I am afraid that they paid little attention to the scientific quality of this article as echoed by the first reviewer as well. Although this is an important information, there are numerous publications on KAP of COVID-19 out there. The present study has many methodological issues that needs to be addressed

1. The main problem which the authors have ignored is that, it is not enough to make sense of descriptive where obviously these were masked by numerous cofounders. In this study, almost all demographic variables were significantly different, one would think that it is clear the results need to adjust for all these. In addition, Authors purport to use statistical inference but their sample is totally biased, meaning that all inference is completely meaningless if not taking serious steps in controlling for bias, refining the population the Authors infer to and keeping interpretations within the realm of relationships between variables rather than characteristics of the population. More sophisticated statistical approaches and substantial analysis to control for biased data is needed
2. Instead of presenting percentages for the outcome variables, the Authors should investigate what relationships hold when controlled for the other variables available. This can adjust for bias. Also, the Authors should reflect upon what relationships do make sense and interpret what they find regarding such reflections. Statistical significance is rather unimportant as it depends on sample size. Control for variables influencing participation in the survey and not predicting the outcome.
3. The outcome of knowledge, attitude and practice should be modelled separately to identify the associated factors.
4. Authors need to explain why the surprisingly high response rate in this study with no incentives and voluntary participation. Intriguing also was the 100% return rate of the survey even though the surveys was distributed via social media (WhatsApp). Firstly, how is it possible that everyone who received an e-link completed the survey and the authors knew they did? Secondly, how did the authors get all hcws email addresses and phone numbers (for those who they received via WhatsApp)? Thirdly, how effective is the use of google form in such surveys and how did the authors deal or avoid double counting?
5. Discussion still does not address the findings, summarise and compare with previous studies. Where they disagree, they need to explore the reason for such differences?
6. Besides controlling for variables measuring selection bias, interaction terms should be investigated. What happens if one crosses sex and region or education and region in predicting knowledge, or risk.
7. The non availability of a third option in most of the studies is problematic particularly the risk perception. For instance, Q2 asked the participant what they think about the severity of the disease. The options were very dangerous and moderately dangerous. How would someone who does not perceive this as being dangerous or simply dangerous respond? Same goes for preventive behaviour: were these questions referring to regular use. This should have been categorised using a Likert Scale.
8. Sample size calculation is incorrect. The referenced study (ref 15) collected data from Iranian University medical students (5-7^th^ year) and was based on a population estimation of 5,000. This is not the same with health care workers in Ankara. What is the population and how did the authors arrive at the sample size used. If all questionnaires were returned and all valid as stated in the method session, assuming every health worker was included, how then was sample size derived. What is the attrition rate of the sampling..
9. It is also important to note that majority of these issues were raised during my initial review and as I can see from reviewer 1’s comment (32 lines of comment), the authors have responded with 4 lines. It is unclear where those changes have been effected. I suggest responding to each of the questions on how they have addressed it will be useful.

**Minor**

Authors should consider graphical presentation of the prevalence and 95% Confidence intervals of the Knowledge, preventive behavior and risk perception scores.

It may be possible to avoid table 1, as the information can all be fitted into the text.

Present a multiples regression table to show unadjusted and adjusted results.
