# Supplementary material for: Knowledge, preventive behaviors and risk perception of the COVID-19 pandemic: A cross-sectional study in Turkish health care workers
Source: PLoS One. 2021 Apr 9;16(4):e0250017. doi: 10.1371/journal.pone.0250017 (PMC8034719; doi:10.1371/journal.pone.0250017)
Supplement: Attachment — Submitted filename: Review_2.docx [file pone.0250017.s005.docx]

Knowledge, preventive behaviors and risk perception of the COVID-19 pandemic: A cross-sectional study in Turkish health care workers

The manuscript still needs further language editing. The mainbody repeated twice and I couldn’t

Some minor concerns are stated below:

Procedure

Page 11- The questionnaire was administered via Google forms in Turkish language. Kindly delete the entire paragraph.

Results

Page 14. Table 1. Kindly rephrase the table as shown below

| Variable | Frequency (%) |
| --- | --- |
| Age (Mean ±SD) |  |
| Sex  Male  Female | 68 (27.1)  183 (72.9) |

Except otherwise requested by the journal, Kindly format all tables to this template. Note the use of horizontal line for each variable and not each option.

Page 15- Paragraph 1- The statement “Table 2 divides…”, change the word divide to describes

Page 15- penultimate paragraph—rephrase the statement to “the rate of use of gloves, medical mask” etc

Is repeating the whole questionnaire appropriate. I would personally suggest moving Table 3 to supplementary table S1.

Discussion

Paragraph 1- Line 5- change breadth of spread to “extent of spread”

Page 24 Paragraph 2- was there a knowledge gap in HCWs in Turkey? If not, rephrase the statement to : “this study assesses the knowledge, PBs and risk perceptions of HCWs in Turkey’s capital city.”
