# Supplementary material for: Knowledge, preventive behaviors and risk perception of the COVID-19 pandemic: A cross-sectional study in Turkish health care workers
Source: PLoS One. 2021 Apr 9;16(4):e0250017. doi: 10.1371/journal.pone.0250017 (PMC8034719; doi:10.1371/journal.pone.0250017)
Supplement: Attachment — Submitted filename: Response.docx [file pone.0250017.s006.docx]

Dear Editor,

We would like to thank again you and the reviewers for their valuable comments and recommendations. On the basis of these, we have revised our manuscript point-by-point response to the comments of the editorial office and reviewers. Our revisions have been highlighted in yellow.

**EDITORIAL OFFICE COMMENTS:**

**Reviewer: 1**

**Comment:** The resubmission of this manuscript has addressed several comments and also improved the language in the text. However, there are still a few typos in the document as well as concerns that the logical translation may not accurately describe the authors intent. There appears to also be a lack of consistency (e.g., on how many decimals are used for percentages) throughout the manuscript. Generally, it reads as though different people wrote different sections. One example of this is that there is no mention that a KAP survey is being implemented in the methods section, though the discussion makes strong mention of a KAP approach.

**Author response:** Thank you for your comment. Due to your request, we have rechecked our manuscript and sending the certificate of Scribendi editing service. Besides, all the decimals consist in the manuscript have been corrected. The article has been re-evaluated in terms of integrity.

**Reviewer: 2**

**Comment:** The main problem which the authors have ignored is that, it is not enough to make sense of descriptive where obviously these were masked by numerous cofounders. In this study, almost all demographic variables were significantly different, one would think that it is clear the results need to adjust for all these. In addition, Authors purport to use statistical inference but their sample is totally biased, meaning that all inference is completely meaningless if not taking serious steps in controlling for bias, refining the population the Authors infer to and keeping interpretations within the realm of relationships between variables rather than characteristics of the population. More sophisticated statistical approaches and substantial analysis to control for biased data is needed

**Author Response:** Our study found the effect of demographic variables on knowledge and preventive behavior to be significant in the analysis with one variable. In line with your suggestions, we examined the effect of demographic characteristics on these scores with Multiple Linear Regression analysis, and the results were showed in Table 4 and 5. The impact of all demographic variables in explaining knowledge and preventive behavior scores were examined with advanced analysis methods. As a result of further analysis, we think we got rid of bias in statistical inferences.

**Comment:** Instead of presenting percentages for the outcome variables, the Authors should investigate what relationships hold when controlled for the other variables available. This can adjust for bias. Also, the Authors should reflect upon what relationships do make sense and interpret what they find regarding such reflections. Statistical significance is rather unimportant as it depends on sample size. Control for variables influencing participation in the survey and not predicting the outcome.

**Author response:** In line with your suggestions, by controlling other variables with advanced analysis methods, which variables affect the scores are examined and given in Tables 4 and 5.

**Comment:** The outcome of knowledge, attitude and practice should be modelled separately to identify the associated factors.

**Author response:** We examined the knowledge and preventative behavior score separately with the Multiple Linear Regression model and given in Tables 4 and 5.

**Comment:** Authors need to explain why the surprisingly high response rate in this study with no incentives and voluntary participation. Intriguing also was the 100% return rate of the survey even though the surveys was distributed via social media (WhatsApp). Firstly, how is it possible that everyone who received an e-link completed the survey and the authors knew they did? Secondly, how did the authors get all hcws email addresses and phone numbers (for those who they received via WhatsApp)? Thirdly, how effective is the use of google form in such surveys and how did the authors deal or avoid double counting?

**Author response:** Thank you for this comment. The pandemic's initiation, the HCW in Ankara, with a number of 340 participants, develop a common WhatsApp group to share the current developments about this pandemic. All the participants shared their email addresses, phone number, and further details to follow the news about this pandemic. Therefore we wrote in the material and method section in lines 90-97. Besides, we have conducted our survey by utilizing google. The main reason is that it was free (no funding required) and easily delivered to every HCWs. The second reason is that we received the participant's data elaborately at the end of the survey without double counting or other complex problems.

**Comment:** Discussion still does not address the findings, summarise and compare with previous studies. Where they disagree, they need to explore the reason for such differences?

**Author response:** We compraded with other studies and cited in the discussion section in line

253, 263-265, 277-279.

**Comment:** Besides controlling for variables measuring selection bias, interaction terms should be investigated. What happens if one crosses sex and region or education and region in predicting knowledge, or risk.

**Author response:** We accept that it would be a correct approach to examine all variables' impact. Estimating the knowledge and preventive behavior with the effect of gender, region, and education were re-examined with advanced analysis methods. They were shown in Tables 4 and 5.

**Comment:** The non availability of a third option in most of the studies is problematic particularly the risk perception. For instance, Q2 asked the participant what they think about the severity of the disease. The options were very dangerous and moderately dangerous. How would someone who does not perceive this as being dangerous or simply dangerous respond? Same goes for preventive behaviour: were these questions referring to regular use. This should have been categorized using a Likert Scale.

**Author response:** In our study, we did not categorize the questions about risk perception to get more precise answers. We aimed to reveal the thoughts on this issue clearly. We did not consider risk perception questions as a scale, so we did not offer Likert type options in categories. We surveyed at a time when the Covid19 pandemic was experiencing a lot of fat. In such a period, it was widespread for all healthcare professionals and the Turkish society that the pandemic was dangerous.

**Comment:** Sample size calculation is incorrect. The referenced study (ref 15) collected data from Iranian University medical students (5-7th year) and was based on a population estimation of 5,000. This is not the same with health care workers in Ankara. What is the population and how did the authors arrive at the sample size used. If all questionnaires were returned and all valid as stated in the method session, assuming every health worker was included, how then was sample size derived. What is the attrition rate of the sampling..

**Author response:** In our study, in order to quickly reveal the knowledge and attitude levels of healthcare professionals during the COVID-19 pandemic period, we accepted healthcare professionals working at the Covid hospital and returning with the answer within one week. We aimed to present the results of the healthcare professionals who evaluated our survey by returning to us in this process where healthcare professionals work intensely. İt would be much better to complete the study with more healthcare professionals, but we thought we had reached a sufficient sample size by calculating the study's power. In the study in which 251 people were included, the statistical power observed for R2 = 0.115 calculated from the multiple regression the power was calculated as 0.99 when the type-1 error rate was taken as 0.05 in the presence of 6 independent variables in the test based on the F statistic of the non-zero difference of the coefficient of determination (R2) in the Multiple Linear regression analysis. The calculation was done using GPower 3.1.9.2 package program. The power analysis results were added as a supplementary file.

**Comment:** It is also important to note that majority of these issues were raised during my initial review and as I can see from reviewer 1's comment (32 lines of comment), the authors have responded with 4 lines. It is unclear where those changes have been effected. I suggest responding to each of the questions on how they have addressed it will be useful.

**Author response:** We have re-evaluated your work in line with the revisions sent by you and other reviews. As a result, our data has changed. New data were re-evaluated using the proposed statistical methods. Multiple regression analysis was performed. According to the first revision, the study's statistics have been improved as much as we can.

**Minor**

**Comment:** Authors should consider graphical presentation of the prevalence and 95% Confidence intervals of the knowledge, preventive behavior and risk perception scores.

**Author Response:** We can obtain numerical data in knowledge and preventive behavior, but we do not gain numerical data in the risk perception due to no scoring system in this part. Therefore we could not draw the appropriate graphical drawing, including all of them.

**Comment:** It may be possible to avoid table 1, as the information can all be fitted into the text.

**Author Response:** Table 1 was deleted as your requested

**Comment:** Present a multiples regression table to show unadjusted and adjusted results. **Author Response:** As you requested, a multiple regression was performed, and the results were illustrated in Tables 4 and 5.

**Comment:** Some minor concerns are stated below:

Procedure

Page 11- The questionnaire was administered via Google forms in Turkish language. Kindly delete the entire paragraph.

**Author response:** The procedure part in the material and methods section was completely deleted as your request.

**Comment:** Results

Page 14. Table 1. Kindly rephrase the table as shown below

| Variable | Frequency (%) |
| --- | --- |
| Age (Mean ±SD) |  |
| Sex  Male  Female | 68 (27.1)  183 (72.9) |

Except otherwise requested by the journal, Kindly format all tables to this template. Note the use of horizontal line for each variable and not each option

**Author response:** Table 1 was erased from the manuscript as your request in major revision. All the tables' horizontal lines were arranged.

**Comment:** Page 15- Paragraph 1- The statement "Table 2 divides…", change the word divide to describes

**Author response:** The sentence was deleted.

**Comment:** Page 15- penultimate paragraph—rephrase the statement to "the rate of use of gloves, medical mask" etc

**Author response:** The sentence was corrected in line 177.

**Comment:** Is repeating the whole questionnaire appropriate. I would personally suggest moving Table 3 to supplementary table S1.

**Author response:** Following this comment, we consider your request, however other manuscript remains this table in the literature and demonstrates the percentage; we prefer to remain this table.

**Comment:**

Discussion

Paragraph 1- Line 5- change breadth of spread to "extent of spread"

**Author response:** The sentence was corrected in line 246.

**Comment:** Page 24 Paragraph 2- was there a knowledge gap in HCWs in Turkey? If not, rephrase the statement to : "this study assesses the knowledge, PBs and risk perceptions of HCWs in Turkey's capital city."

**Author response:** As a result of the multiple regression analysis, when all variables were evaluated, there were differences regarding knowledge, preventive behavior, gender, profession. The differences were stated in the text in line 277-279. Furthermore, there was not a knowledge gap in Turkey according to the results.

**Reviewer: 3**

**Comment:** I have reviewed the manuscript titled "Knowledge, Attitudes and Practices of Turkish Healthcare Workers About SAR2-CoV-19 During Pandemic" submitted to "PlosOne" for publication. In this paper, authors have conducted a survey study to investigate the level of COVID-19 related knowledge in health care workers in Turkey. This is a well conducted study and the manuscript is well presented and fits within the scope of the journal; it needs some major improvements; there are a few suggestions that authors may consider improving it further:

The use of English language is reasonable, however, there are a number of punctuation and grammatical errors; that should be corrected and rephrased using academic English for a better flow of text for reader. Authors may consider proofreading this manuscript.

**Author Response:** In accordance with this comment, the language has been revised again by a native English speaker. The punctuation and grammatical errors were rechecked.

**Comment:** Line 28: "In"?

**Author Response:** Line 28 'ın' was rechecked and corrected.

**Comment:** I have no more comments regarding the introduction, methods and results as all sections have been well presented. The discussion section can be further improved. Surprisngly, authors missed inclusion of many recent studies that are similar to the subject. Here are a few examples;

COVID-19 Pandemic and Role of Human Saliva as a Testing Biofluid in Point-of-Care Technology. European Journal of Dentistry. 2020 Jun 3.

Knowledge and Attitude of Dental Practitioners Related to Disinfection during the COVID-19 Pandemic. Healthcare 2020, 8, 232.

Fear and practice modifications among dentists to combat Novel Coronavirus Disease (COVID-19) outbreak. International Journal of Environmental Research and Public Health. 2020 Jan;17(8):2821.

Author may discuss finding of such studies in context to improve the discussion.

**Author response:** Knowledge and Attitude of Dental Practitioners Related to Disinfection during the COVID-19 Pandemic. Healthcare 2020, 8, 232. and Fear and practice modifications among dentists to combat Novel Coronavirus Disease (COVID-19) outbreak. International Journal of Environmental Research and Public Health. 2020 Jan;17(8):2821 were cited the appropriate parts in the manuscript in line 253 and line 263, respectively.

**Comment:** There are a number of statements in the discussion require reference citations; please make sure, appropriate citations are in place.

**Author Response:** Appropriate citations were added to the manuscript.

**Comment:** What authors' suggestions to further improve the level of COVID-19 related Knowledge among HCW?

**Author Response:** We make the suggestion in conclusion part line 323-328.

**Comment:** Line 308: educatşonal? Please correct

**Author Response:** Line 308, 'educatşonal?' was corrected.

**Reviewer 5**

**Comment:** There are methodological issues with the current manuscript and some reports of 100% survey returned despite using online distribution platform, lack of incentive and participation was voluntary and no professional body helped with providing emails of members is unusual.

**Author Response:** Thank you for your special comments. Sorry for this misunderstanding. We have forgotten the total number of HCW as 340; however, 251 of HCW have sent appropriate answers. Therefore, we have arranged the correct form and mentioned it in the material method section, line 90-97.

On behalf of all of the authors, I would again like to thank the reviewers for their valuable comments and recommendations.

Thank you for your attention.
